# Vestibular attenuation to random-waveform galvanic vestibular stimulation during standing and treadmill walking

**DOI:** 10.1038/s41598-021-87485-4

**Published:** 2021-04-14

**Authors:** Kelci B. Hannan, Makina K. Todd, Nicole J. Pearson, Patrick A. Forbes, Christopher J. Dakin

**Affiliations:** 1 Department of Kinesiology and Health Science, Utah State University, Logan, UT USA; 2 Department of Neuroscience, Erasmus MC, University Medical Center Rotterdam, Rotterdam, The Netherlands

**Keywords:** Motor control, Sensorimotor processing, Sensory processing

## Abstract

The ability to move and maintain posture is critically dependent on motion and orientation information provided by the vestibular system. When this system delivers noisy or erred information it can, in some cases, be attenuated through habituation. Here we investigate whether multiple mechanisms of attenuation act to decrease vestibular gain due to noise added using supra-threshold random-waveform galvanic vestibular stimulation (GVS). Forty-five participants completed one of three conditions. Each condition consisted of two 4-min standing periods with stimulation surrounding a 1-h period of either walking with stimulation, walking without stimulation, or sitting quietly. An instrumented treadmill recorded horizontal forces at the feet during standing and walking. We quantified response attenuation to GVS by comparing vestibular stimulus-horizontal force gain between conditions. First stimulus exposure caused an 18% decrease in gain during the first 40 s of standing. Attenuation recommenced only when subjects walked with stimulation, resulting in a 38% decrease in gain over 60 min that did not transfer to standing following walking. The disparity in attenuation dynamics and absent carry over between standing and walking suggests that two mechanisms of attenuation, one associated with first exposure to the stimulus and another that is task specific, may act to decrease vestibulomotor gain.

## Introduction

Motion and orientation information encoded by the vestibular system is critical for our ability to interact with the world. When this information is erroneous or irrelevant to our behavioral goals, the nervous system may attenuate the behavioral response to the information over prolonged or repeated presentations, often through a process known as habituation^1,2^. Stimulus-related vestibular attenuation can present in a variety of ways, such as a decreasing gain or advancing phase of the vestibulo-ocular reflex in response to repeated rotation or caloric stimuli (Rotation^1,3–7^, Caloric^8–10^), a decrease in rotation sensation following repeated rotations^11^, and decreased responsiveness to prolonged or repeated bouts of galvanic vestibular stimulation (GVS)^12–17^.

In humans, reports of stimulus-related vestibular response attenuation to repeated bouts of GVS have been inconsistent. During standing, a rapid decrease in GVS-induced postural sway has been observed during the first 40 s of exposure to sinusoidal stimuli^12^, but no further response attenuation is observed with repeated exposure to the stimulus. In contrast, response attenuation continues across repeated exposures to random-waveform (‘noisy’) GVS in participants performing a battery of tasks (including posturography assessment, vestibulo-ocular reflex testing, and walking)^15^. An important difference between these observations may be the different tasks posed to the participants of each study. In the first study, participants stood for the entire trial, and in the second study, participants walked some of the time. Therefore, the differences in the rate and duration of vestibular response attenuation between these studies could arise from task-dependent mechanisms, as reported previously^18^.

Here we sought to determine whether prolonged exposure to supra-threshold random-waveform GVS results in task-dependent stimulus-related vestibular response attenuation during standing and walking. Participants stood, with stimulation, for 4 min. Then, for 60 min, the participants either walked with stimulation, walked without stimulation, or sat without stimulation, followed by a second 4-min standing period with stimulation. During standing, we hypothesized a small, rapid stimulus-related vestibular response attenuation that would saturate in the first 40 s of stimulation, as reported previously by Balter et al.^12^. If there is a walking-related attenuative mechanism^18^, we then expected to observe a continuation of the decrease in the magnitude of vestibular responses once walking started, but with a different saturating time constant than observed during standing. We found that during standing, vestibular gain decreased only during the first 40 s, with a time constant of 19 s. This decrease in gain however, was insufficient to produce a statistical difference in vestibular gain between standing periods before and after walking. In contrast, during walking we observed a significant decrease in vestibular gain (~ 38%) that continued throughout the 60 min walking period (time constant of 67 s). Since the stimulus-related vestibular response attenuation observed during walking did not carry over into the final standing period the mechanism appears walking-related.

## Methods

### Participants

Fifty-five participants (21 male, 34 female) were recruited from the university campus. Forty-five were included in the final analysis (18 males, 27 females, 22.1 ± 2.9 years, 172.2 ± 8.4 cm, 70.5 ± 15.1 kg) due to three participants not completing the study and exclusion of seven participants’ data due to equipment failure (i.e. damaged heel sensor) or data collection error (i.e. key signals missing in the recording). Participants had no known history of neurological disease and each provided informed, written consent prior to participation. All procedures conformed to the declaration of Helsinki and were approved by Utah State University’s Institutional Review Board (protocol #9395).

### Procedures

**Figure 1:**
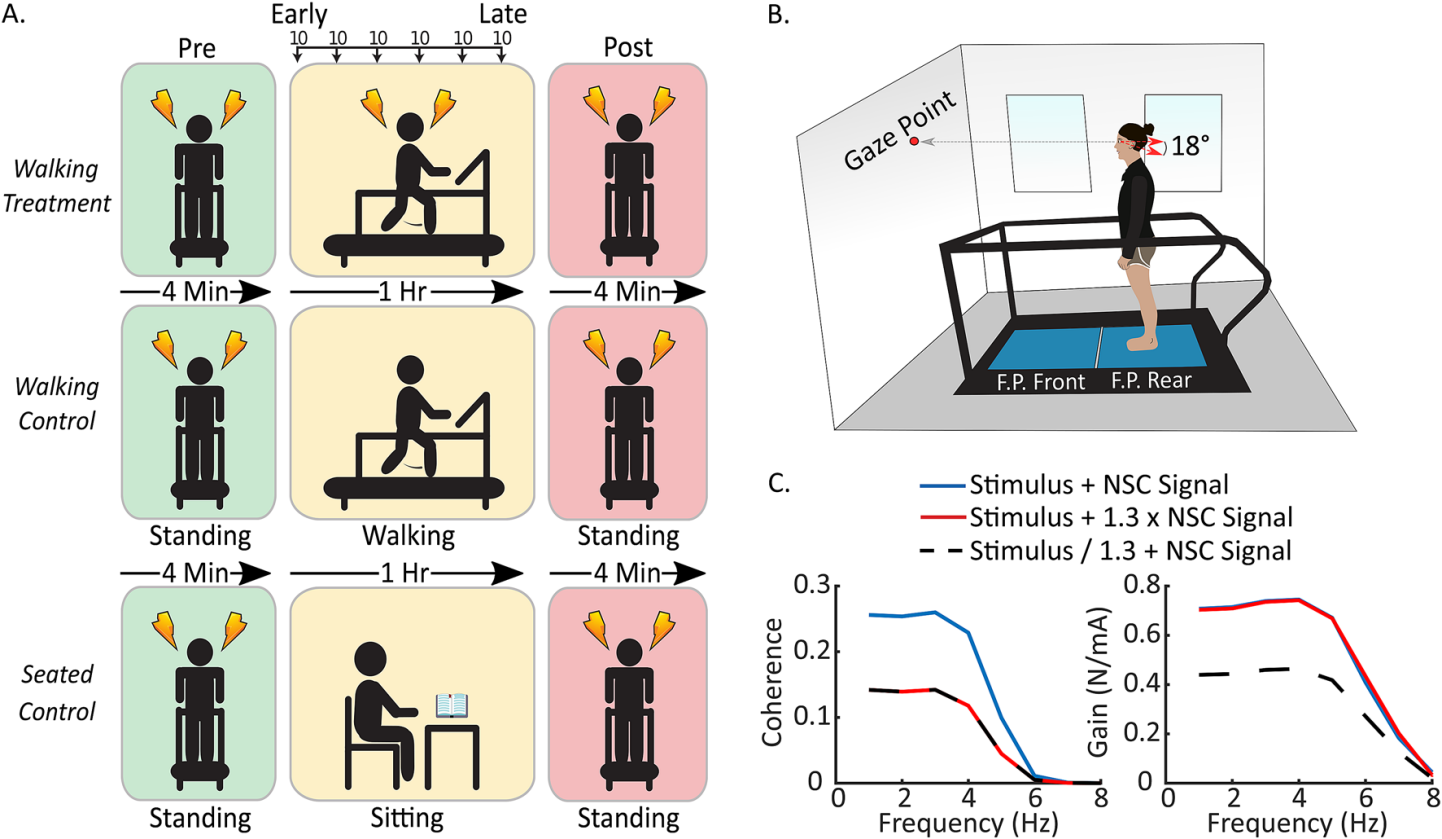
Experimental protocol completed by the three procedural groups, experimental setup, and predicted results. (**A**) Each group stood for 4 min at the start and end of the experiment while receiving galvanic vestibular stimulation. Between the two 4-min standing periods, the *Walking Treatment* group walked for six consecutive 10-min periods while receiving stimulation, the *Walking Control* group walked for 60 min without stimulation, and the *Seated Control* group sat and read for 60 min without stimulation. (**B**) Participants stood or walked on the rear force plate of a tandem two-belt instrumented treadmill. They fixated at a point on the wall to maintain their head orientation at Reid’s plane tilted 18° nose up. (**C**) Simulation of likely potential outcomes. The left panel illustrates changes in coherence relative to baseline (blue line) when non-stimulus correlated (NSC) signal increases relative to baseline (red line) or when the body habituates to the stimulus, decreasing the amplitude of the stimulus-correlated signal relative to non-stimulus correlated signal (black segmented line). The right figure illustrates prospective changes in gain. With pure stimulus-related response attenuation, we expect both coherence and gain to decrease (black segmented line). If non-stimulus correlated signal increases relative to stimulus correlated signal, we expect coherence to decrease but gain to remain similar (red line) compared to baseline (blue line).

Upon arrival at the testing facility, participants were screened for physical capability using a Physical Activity Readiness Questionnaire (PAR-Q) and a GVS Pre-Screening Questionnaire (Supplementary Methods S1). After meeting the participation requirements, participants were assigned to one of three groups: *Walking Treatment* (6 males, 9 females, 22.9 ± 2.6 years), *Walking Control* (6 males, 9 females, 21.4 ± 1.8 years)*,* and *Seated Control* (6 males, 9 females, 21.9 ± 3.8 years). The *Walking Treatment* group served as the intervention group to quantify stimulus-related vestibular response attenuation during standing and walking. The *Walking Control* group sought to determine whether pre-post changes in the *Walking Treatment* group were due to stimulus exposure during walking. Lastly, the *Seated Control* group sought to determine if walking itself, without stimulation, altered participant behavior in the post-standing trial. Participants were initially pseudo-randomly assigned to the *Walking Treatment* and *Walking Control* groups, such that if a participant was assigned to the treatment group, the next participant was assigned to the control group and vice-versa. Following collection of the *Walking Treatment* and *Walking Control* groups, we determined that an additional *Seated Control* group was necessary to assess the influence of walking on the pre and post standing trials in the *Walking Treatment* and *Walking Control* groups (Fig. 1). Therefore, an additional fifteen subjects were collected.

A wireless force transducer (Delsys Trigno, Natick, MA, USA) was placed on the left heel of all participants to record heel strike for each stride and to maintain consistency between conditions in each group. Two conductive gel coated carbon–rubber electrodes (3.8 cm × 4.4 cm, Covidien Uni-patch, Dublin, IE) were placed bilaterally over all participants’ mastoid processes to pass the electrical stimulus, generated in Labview^19^, from the Biopac STMISOLA stimulator (Biopac Systems, Inc, Goleta, CA, USA) to the vestibular nerves. Although the skin beneath the electrodes was not cleaned prior to electrode placement nor was the impedance measured, the stimulator maintained a constant current for all participants ensuring that the same current stimulus was delivered across participants. The direction of GVS-elicited postural responses depends on the relative orientation of the head and feet^20^; therefore, we made two efforts to restrict the postural response, as much as possible, to the frontal plane^21–23^ by controlling head orientation relative to the body and feet. First, we placed two stickers on the left side of each participant’s head, one at the corner of the eye and one behind the ear 18° above Reid’s plane (the line from the eye to the external auditory meatus^22^), so that researchers could visually monitor and control each participant’s head pitch during the experiment (Fig. 1B). If the line intersecting the two stickers tilted from horizontal, the experimenter would instruct the participant to tilt their head up or down, to bring this line back to horizontal. Additionally, during standing and walking, participants gazed at a dot on the wall in front of them to help maintain the desired head orientation. Prior to data collection, each participant received two 2 s periods of stimulation with peak amplitudes of 3 mA and 5 mA while seated, to experience the stimulus and indicate their willingness to proceed with the experiment. No participants were unwilling to start the experiment. For the rest of the experiment participants received a bandwidth limited 0–25 Hz random noise peaking at 5 mA^24^. Stimuli were generated by low-pass filtering a white noise signal using a 4th order butterworth filter, with cutoff of 25 Hz. The signal was then rescaled to a peak amplitude of 5 mA.

The experimental protocols for each group were similar (Fig. 1A). Participants began by standing on the back force-plate of a two force-plate instrumented treadmill (AMTI, Watertown, MA, USA; Fig. 1B) for 4 min, with their head facing forward, eyes open, and feet together (medial malleoli touching), while receiving random-waveform GVS. The treadmill surface was firm, providing a walking and standing surface similar to a hard floor. The instrumented treadmill contained two force plates, arranged front-back, each measuring three-dimensional forces and moments. This first standing period we label as the ‘pre’-standing trial because it was completed before each group performed a separate task for 60 min. The *Walking Treatment* group walked on the back force-plate of the instrumented treadmill for six 10-min stimulation blocks, at a metronome-synchronized cadence of 76 bpm. Cadences at this approximate rate have been previously used effectively to examine vestibular influence during walking^25–27^. The 60-min period was broken into 10-min intervals to reduce data file size. We defined the first 10-min period of walking as ‘early’ walking and the last 10-min walking period as ‘late’ walking, to distinguish their comparison from the surrounding standing periods. Between each 10-min period, participants continued to walk while researchers re-started data collection and the stimulus. The *Walking Control* group walked similarly to the *Walking Treatment* group but without stimulation. The *Seated Control* group sat and read a book for 60 min. We did not restrict their head orientation while reading because they were neither being stimulated nor walking. Following the 60-min block, all participants completed a second 4-min ‘post’-standing trial, which was identical to the pre-standing trial, but was labelled as post-standing trial as it followed the walking or sitting period.

### Data analysis

To quantify vestibular attenuation, we first estimated the coherence and gain between the input electrical stimulus (GVS in mA) and the output medio-lateral horizontal force response, as measured by the rear force plate in the treadmill (Newtons), assuming a linear stimulus–response relationship^28^. Coherence measures the linear relationship between two signals. It ranges from zero to one (zero is no relationship and one is a perfect linear relationship with no noise), and it indicates the proportion of variance shared between two time-series at a given frequency^29^. We used coherence to assess the relative contribution of vestibular signals to the evoked balance response. We used gain to quantify the ratio of the output signal (lateral forces at the feet) to the input signal (GVS signal). A gain greater than one indicates that the output signal at a given frequency is greater than the input signal. We expected stimulus-related vestibular response attenuation to present as a decrease in the amplitude of stimulus-correlated forces relative to the stimulus amplitude, which remains constant. Coherence and gain should therefore decrease (Fig. 1C), provided non-vestibular drive to the muscle remains constant. However, coherence could decrease but gain stay the same if non-vestibular drive to the muscle increases over time while the stimulus-correlated output stays the same (i.e. no vestibular response attenuation) (Fig. 1C). Standing and walking trials were analyzed separately offline using custom Matlab scripts (Mathworks, Natick, MA, USA).

### Standing: General vestibular response attenuation

To identify stimulus-related vestibular response attenuation over the duration of the experiment, we first compared vestibulomotor coherence and gain for the entire 4-min pre and post-standing trials. Coherence and gain for these trials were calculated using the Neurospec Matlab library (http://www.neurospec.org/) based on the method described by Rosenberg et al.^30^. The library provides a 95% confidence limit for the coherence estimate based on the number of disjoint segments contributing to the coherence estimate. Pre and post-standing coherence and gain were compared at frequencies in which coherence was significantly different from zero.

**Figure 2:**
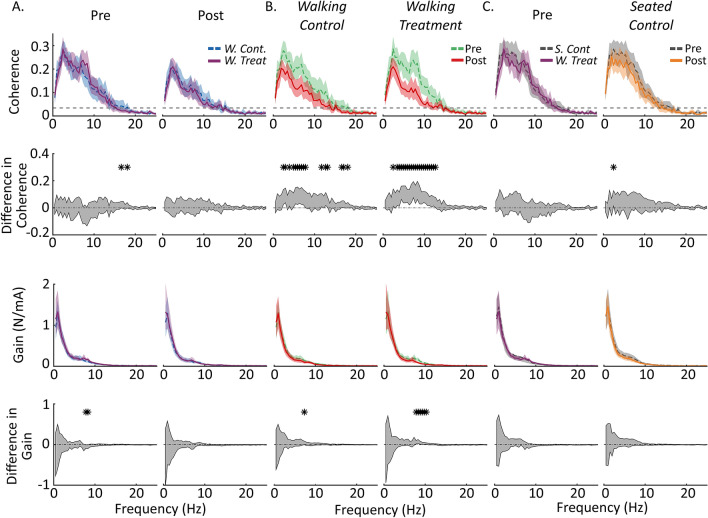
Coherence spectra and 95% confidence intervals for each of the standing conditions and their statistical comparisons. (**A**) Coherence and gain spectra for the pre and post-standing periods. The blue segmented line is the *Walking Control* condition, and the violet solid line is the *Walking Treatment* condition. Both before and after walking the coherence and gain spectra for the two conditions are not broadly statistically different. The segmented lines in the upper panels (coherence) are the 95% confidence limits. Coherence lower than these limits were not significantly different than zero. (**B**) Coherence and gain spectra compared pre and post within the *Walking Control* and *Walking Treatment* conditions. The green segmented lines are the pre-standing periods and the red solid lines are the post-standing periods. After walking, coherence decreased regardless of whether participants received stimulation. Gain did not exhibit any broad differences between pre and post. (**C**) The first column is the pre-standing period for the *Seated Control* (gray segmented line) compared to the pre-standing period for the *Walking Treatment* condition (violet solid line). There were no broad significant differences between the two groups at the start. The second column in (**C**) compares the pre and post-standing periods for the *Seated Control* condition. No broad significant differences existed between the two standing periods in the *Seated Control* condition. Each curve n = 15, and the difference in coherence and gain (row 2 and row 4) was evaluated by bootstrapping the difference in means between the two coherence distributions and determining the 95% confidence interval for the difference in mean distribution (gray segmented line surrounding the shaded region). Regions where the 95% confidence interval for the difference in means excluded zero were deemed significantly different and have been indicated with a star.

To illustrate the pre and post-standing coherence and gain (Fig. 2), we bootstrapped 95% confidence intervals for the mean at each frequency in both measures using the percentile method^31^. Fifteen participants’ data were drawn randomly, with replacement, from the empirical sample to form the bootstrap sample, from which a bootstrap sample mean was calculated. This process was repeated ten-thousand times. At each frequency, the data were ordered and the 250th and 9750th data point taken as the lower and upper confidence limit. The grand mean and confidence intervals are shown in Fig. 2 (Rows 1 and 3).

For statistical comparison of standing coherence and gain before and after walking (during pre and post-standing trials), we applied an extended procedure similar to that used above in calculating coherence and gain. First, 15 participants’ data were drawn randomly, with replacement, from the empirical pre and post-standing sample. We then calculated the mean for both bootstrapped samples (e.g. pre and post-standing) and subtracted one mean from the other. This process was repeated ten thousand times to estimate the pre and post-standing data’s difference-of-means distribution. The difference-of-means distribution was then ordered at each frequency and the 95% confidence interval determined by taking the 250th and 9750th data point. Regions in the coherence and gain spectra where the 95% confidence interval excluded zero were deemed significantly different from each other (Fig. 2, Rows 2 and 4).

Since we observed a decrease in coherence between the pre and post-standing trials but little decrease in gain (see Results), we sought to determine if the decrease in coherence was due to an increase in horizontal force variability related to the extended period of walking rather than the GVS. To assess this, we calculated the root mean square (RMS) of the medio-lateral forces and compared these values between the pre and post-standing trials of each of the three conditions using Bonferroni corrected t-tests (p < 0.05/3).

### Standing: Rapid vestibular response attenuation

To determine if a brief, rapid stimulus-related vestibular response attenuation occurred at the start of the standing trials^12^, we analyzed the data a second time using higher resolution methods. Rather than using the entire 4-min period, as described above, we calculated the mean gain for each 20-s period in the 4-min pre and post-standing trial of each condition. We combined the data for the pre-standing trials of all 45 subjects because there were no experimental differences between groups at this point. We separated the post-standing trials for each group, since they each performed a different task in the hour between standing trials. To determine the rate of response attenuation over time, we fit the change in mean gain over time with three models: an intercept only model, a linear model, and a decaying exponential. We ultimately estimated the time constant of the exponential model as the time taken to reach 63% of its starting value. The decaying exponential was of the form:

$$y=a{e}^{-bx}+c,$$

where a, b and c are constants, x is a time vector, and $e$ is an exponential. Models were compared using Akaike Information Criterion (AIC) and Bayesian Information Criterion (BIC), where in each measure a lower (or more negative) number indicates a better fit.

### Walking: General vestibular response attenuation

To reveal walking-related vestibular response attenuation, we compared coherence and gain for the early (first 10 min) and late (last 10 min) walking periods in the *Walking Treatment* group. Data were first cut into strides synchronized on the left heel strike using the force sensing resistor placed on the foot. Heel contact was identified visually using a custom Matlab script. Each segment was padded with an additional 25% of the preceding and subsequent stride to prevent coherence and gain distortion at the start and end of the stride. Then the data were low-pass filtered at 30 Hz using a 4th order zero phase shift Butterworth filter and down-sampled to 200 Hz. Step data for the horizontal forces and stimulus were converted to the frequency domain using a Morlet wavelet transform following a modified version of the methods outlined by Zhan et al.^32^. To account for stride-to-stride variability, stride duration was normalized in time by resampling the cross and auto-spectra to the average stride length^25,26^. Time-normalized time-dependent coherence and gain were then averaged over steps within each subject to provide one time-frequency coherence and gain estimate for each subject.

We compared time-dependent coherence and gain for the early and late-walking periods using similar bootstrapping procedures to the pre and post-standing comparison but applied to each point in time and frequency. Based on the number of steps analyzed, coherence during walking was only significantly different from zero over the frequency bandwidth of 0–10 Hz. Therefore, we only compared coherence and gain over this bandwidth.

### Walking: Trend over time

To evaluate the change in coherence and gain over time, we calculated the mean (across time and frequency) coherence and gain, only where coherence was significantly different from zero, in each 10-min period. Comparisons were performed only where significant coherence exists because estimates of gain become unreliable when no linear relationship (significant coherence) exists between the input and output^33^. We then estimated the time constant of the decay in mean gain over the six 10-min walking periods by fitting an exponential, of the same form as that used for the standing data, to the grand mean walking data for each subject and estimated its time constant. Changes in medio-lateral force variability over the hour of walking were examined by subtracting the mean medio-lateral force for each step from each step to center the forces around zero. Step lengths were resampled in time to the mean step length within each subject and then the mean medio-lateral force profile for all steps in each subject was subtracted from each individual step to get the difference of each step from each subjects’ mean step. The RMS of the difference in force from the mean was calculated in each subject at each time point and compared visually between the early and late walking periods.

## Results

### Limited stimulus-related vestibular response attenuation during standing

#### Pre-standing trials

Baseline coherence and gain measures during the pre-standing trials were comparable between the three groups (Fig. 2A,C). Individual subjects’ standing coherence peaked with magnitudes between 0.26 and 0.37 across all groups (*Walking Treatment* pre: 0.37 ± 0.08, post: 0.26 ± 0.07; *Walking Control* pre: 0.35 ± 0.07, post: 0.27 ± 0.07; *Seated Control* pre: 0.37 ± 0.08, post: 0.35 ± 0.1) and dropped to near zero at frequencies greater than 15 Hz (Fig. 2, top row). Standing gain, in contrast, peaked with magnitudes between 1.33 and 1.57 N/mA (*Walking Treatment* pre: 1.40 ± 1.00 N/mA, post: 1.48 ± 1.20 N/mA; *Walking Control* pre: 1.33 ± 0.58 N/mA, post: 1.35 ± 0.80 N/mA; *Seated Control* pre: 1.57 ± 0.85 N/mA, post: 1.57 ± 0.75 N/mA) (Fig. 2, Rows 3 and 4). Generally during the pre-standing trials, there were no broad significant differences between groups over the region of significant coherence (0–15 Hz) in either standing coherence or standing gain (Fig. 2A, Rows 2 and 4).

#### Post-standing trials

Following the one-hour walking period, post-standing coherence decreased in both walking groups (*Walking Treatment* and *Walking Control*), compared to pre-standing coherence values (Fig. 2B, Row 2). The average decrease in standing-coherence was between 0.04 and 0.05 (*Walking Treatment:* 0.05 ± 0.03 and *Walking Control:* 0.04 ± 0.03) and was significant at most frequencies with substantial coherence (i.e. 2–15 Hz) (Fig. 2B, Row 2). Standing-gain decreased comparatively little due to walking, with the only significant decreases occurring at a limited number of frequencies (i.e. 7–11 Hz) in the *Walking Treatment* group (Fig. 2B, Row 4). To determine if stimulus exposure during walking resulted in additional vestibular response attenuation during standing, we compared standing coherence and gain between the post-standing trials of the *Walking Treatment* and *Walking Control* groups. There were no statistical differences over the region of prominent coherence (0–15 Hz) between the post-standing trials of the *Walking Treatment* and *Walking Control* groups after the hour of walking, suggesting that the changes in standing coherence were not due to the hour of stimulation (Fig. 2A, Column 2).

To investigate the coherence decrease observed between the pre and post-standing trials further, we compared the coherence and gain for the *Seated Control* pre and post-standing trials, to determine if the decrease in standing coherence observed in the walking conditions was due to factors related to walking. There was no statistical decrease in coherence in the pre and post-standing trials of the *Seated Control* condition, suggesting the decrease in coherence observed in the *Walking Treatment* and *Walking Control* groups was indeed due to factors related to walking (Figs. 2C, Rows 2 and 4).

### Early rapid stimulus-related vestibular response attenuation

**Figure 3:**
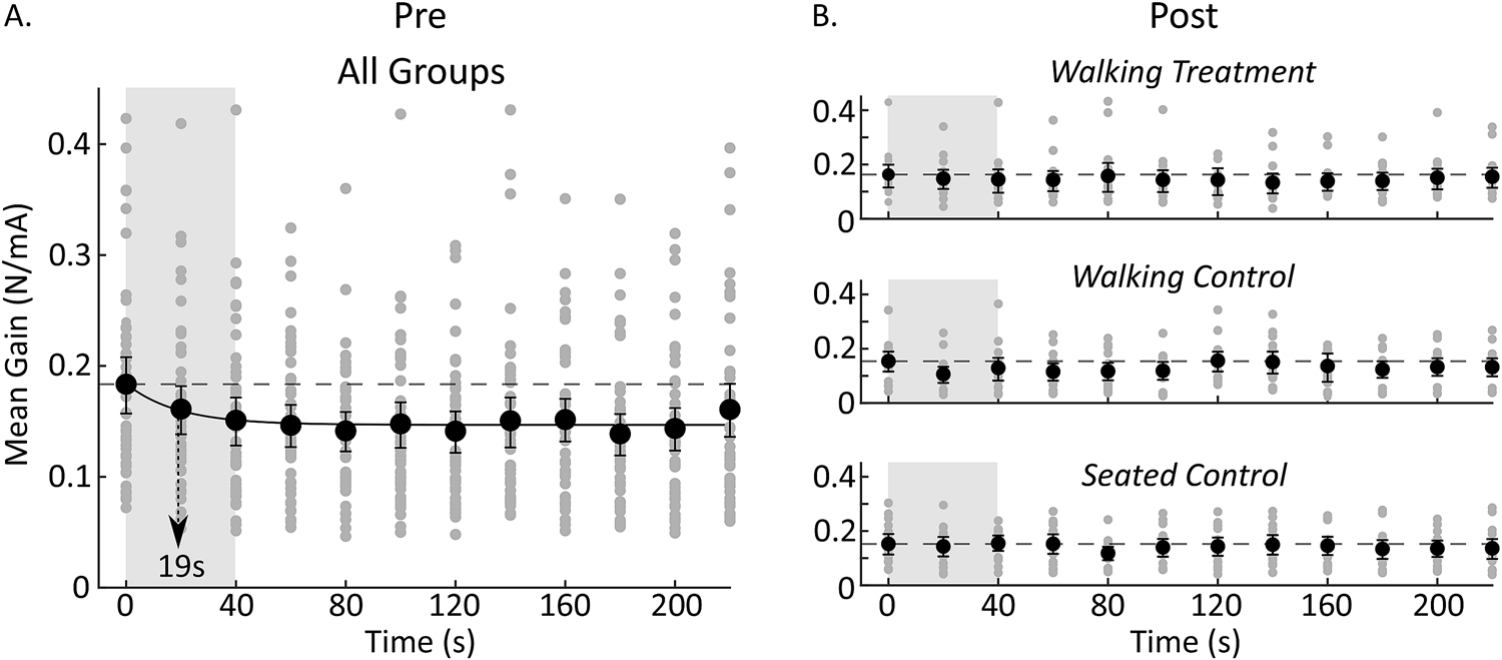
Changes in mean gain over time in both the pre and post-standing periods calculated using twenty seconds of data every 20 s over the 4-min trial. (**A**) Mean gain for the pre-standing period aggregated across all groups (because each condition was similar at this point). Mean gain decreased following a decaying exponential with a time constant of 19 s (n = 45). Arrow indicates the exponential’s time constant. (**B**) Mean gains for the post-standing periods. There were no apparent decreases in mean gain over time in the post-standing period of any of the conditions. Segmented horizontal line serves as a reference indicating the amplitude of the first time point. Error bars are 95% confidence intervals for the mean.

To determine whether small, rapid stimulus-related response attenuation occurs at the start of stimulation, we estimated gain for each 20-s interval over each of the standing periods for each subject in each group. We observed a roughly 18% decrease in gain over the first 40 s of the pre-standing trial and a small decrease in coherence over the full 4-min period. We fit the decay in standing gain over time with an intercept only model (AIC: − 70.48, BIC: − 82.89), a linear model (AIC: − 73.28, BIC: − 72.31), and an exponential model (AIC: − 89.26, BIC: − 88.29), with the exponential model providing the best fit (parameters for the best fit are shown in Table 1 upper half). The decay time constant for the exponential decrease in standing gain was approximately 19 s (Fig. 3A). This small rapid change in gain, however, was not sufficient to produce a broad significant pre and post-standing difference in gain for any of the standing conditions (Fig. 2, row 4). In addition, there were no signs of rapid stimulus-related response attenuation in the post-walking standing period of any condition (Fig. 3B). However, it is important to note that we did not pool the data for the post-standing tasks because of the different tasks performed by each group during the 60-min period prior. The absence of rapid stimulus-related response attenuation in the post-walking standing task could therefore be due to a lack of statistical power, rather than evidence that rapid response attenuation was not observed following walking. It is also unclear whether the rapid response attenuation in the pre-walking standing condition is retained across the different tasks or present in the walking task.

**Table 1:** Statistics for the parameter estimates of the two best fit exponential models.

Final model: mean gain = a*exp(− b*x) + c				
Parameter	Estimate	Stand. Err	t-Stat	p value
**Pre-standing**				
a	0.037	0.0068	5.479	0.0004
b	0.054	0.0239	2.25	0.051
c	0.147	0.0023	63.598	2.97 × 10^–13^
**Walking**				
a	2.0396	1.2698	1.6063	0.2066
b	0.01499	0.0174	0.8598	0.4531
c	0.903	1.4365	0.6286	0.5742

The first is during standing and the second during walking.

### Pre-post standing coherence changes due to factors other than vestibular response attenuation

**Figure 4:**
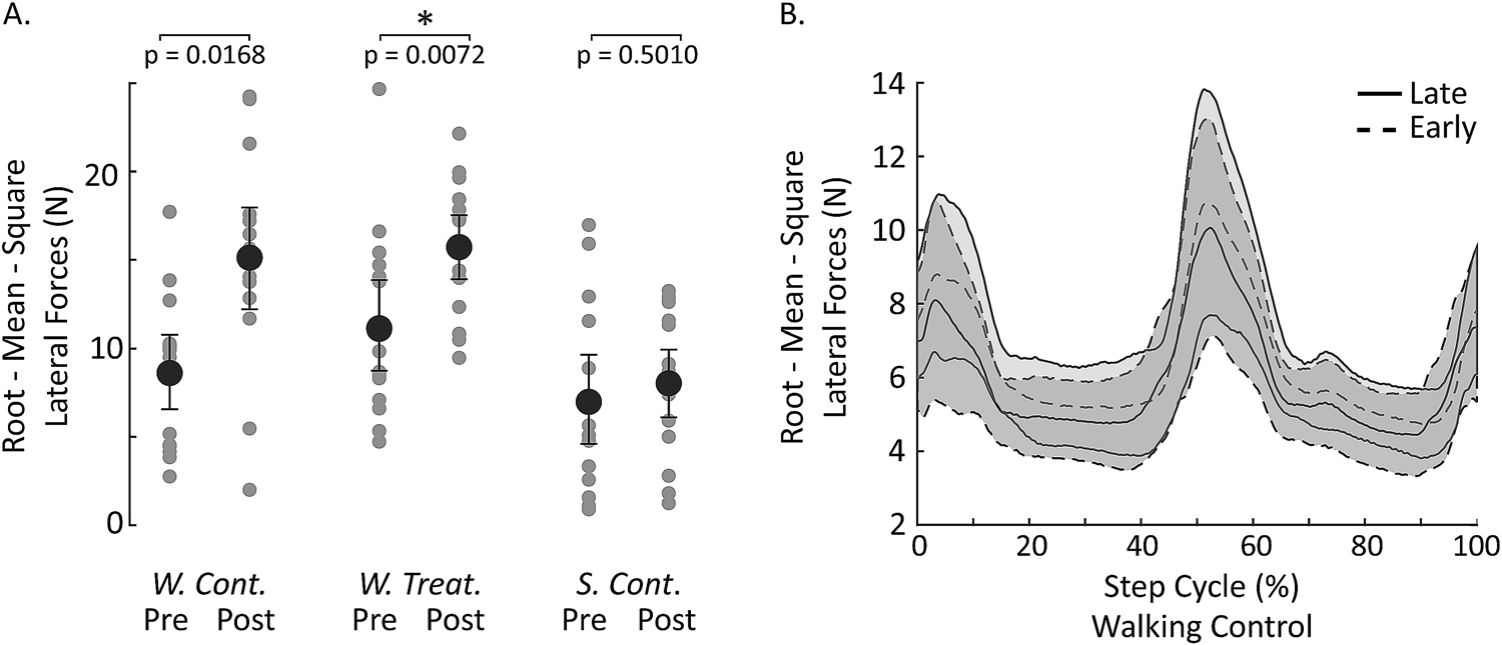
Medio-lateral force variability during standing and walking. (**A**) Root-mean-square (RMS) medio-lateral forces increase following walking (significantly so after the treatment condition) but not following an hour of sitting quietly (n = 15 for each group). *W. Cont.* walking control, *W. Treat.* walking treatment, *S. Cont.* seated control. Pre-post comparisons were performed using Bonferroni corrected t-tests, p < 0.05/3. Statistical significance indicated by *. Error bars are 95% confidence intervals for the mean. (**B**) Variability about the mean RMS medio-lateral forces during walking did not appear to change over time. Shaded area is the 95% confidence interval for the mean. The data for (**B**) were low-pass filtered at 20 Hz to remove high frequency noise from the treadmill belt.

The pre and post-standing trials exhibited a significant difference in coherence that did not appear to be due to the stimulus, as it was also present when participants walked for an hour without stimulation. Therefore, we sought to determine whether the decrease in standing coherence was caused by a relative increase in medio-lateral force variability unrelated to the stimulus, resulting in a decrease in the proportion of overall variability attributed to the stimulus (Fig. 1C: red line). Indeed, RMS medio-lateral forces increased in the post-standing trials compared to the pre-standing trials in both the *Walking Treatment* (pre: 8.6 ± 4.3, post: 15.1 ± 6, p = 0.0072) and *Walking Control* (pre: 11.1 ± 5.3, post: 15.7 ± 3.8, p = 0.0168) groups*,* though only significantly in the *Walking Treatment* group. In comparison, there was no change in RMS medio-lateral forces in the *Seated Control* group (pre: 6.9 ± 5.2, post: 8.0 ± 4.0, p = 0.501) (Fig. 4A). These results suggest that decreases in standing coherence observed during the *Walking Treatment* and *Walking Control* conditions are due to an increase in medio-lateral force variance not linearly related to the stimulus (GVS) and perhaps associated with prolonged walking.

### Stimulus-related vestibular response attenuation during locomotion

**Figure 5:**
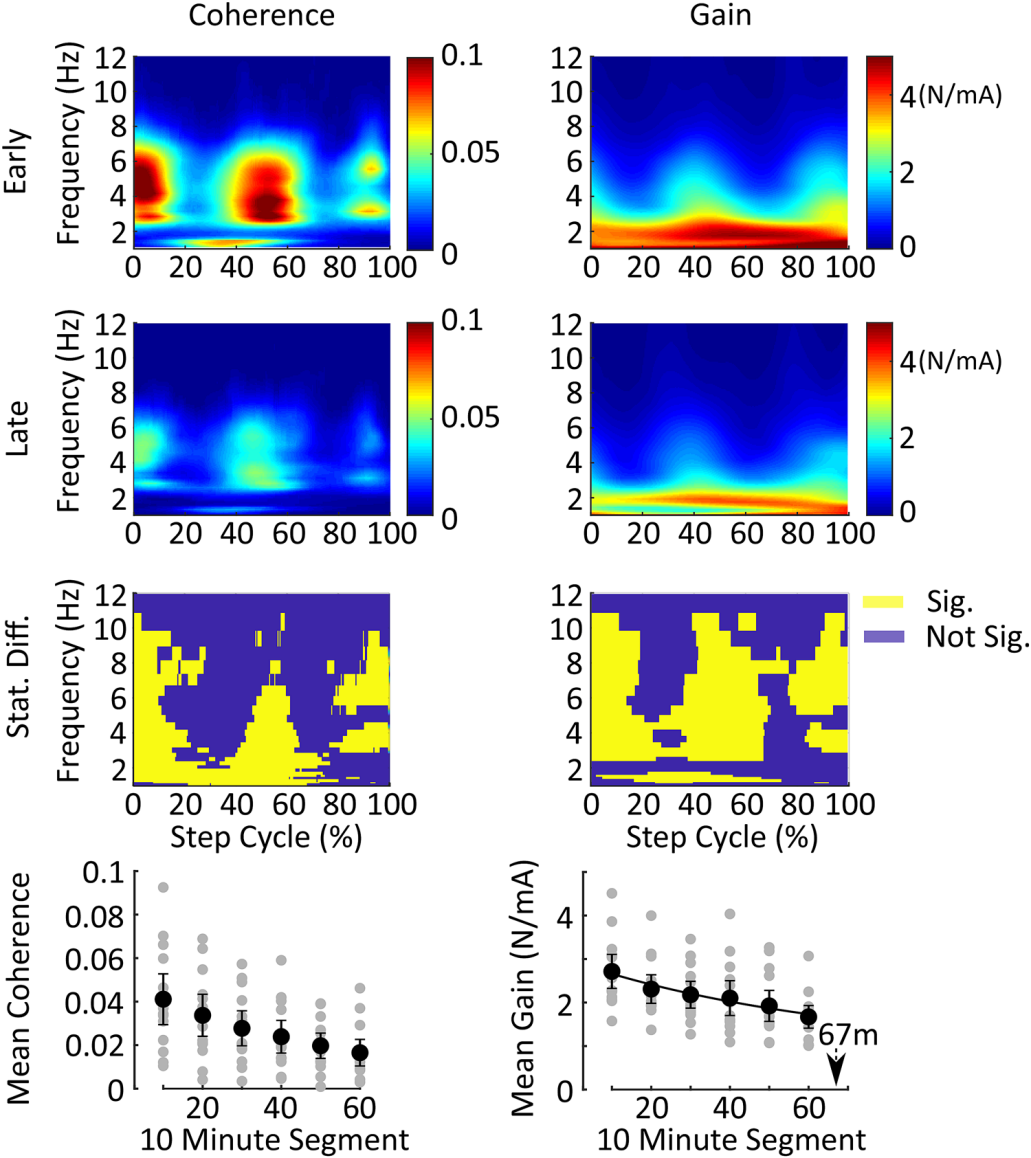
Time–frequency representation of the change in stimulus-medio-lateral force coherence and gain during each stride of the *Walking Treatment* condition. The top three rows illustrate changes in walking-coherence (first column) and walking-gain (second column) for the first (early) and last (late) 10-min walking periods. Prolonged periods of stimulation during walking resulted in a significant decrease in walking-coherence and walking-gain (row 3). The difference in coherence and gain plots were calculated by bootstrapping the difference-of-means distribution over time and frequency, and the 95% confidence interval was estimated using the percentile method. Regions in the early and late panels where the confidence intervals of the difference-of-means distribution excluded zero (yellow regions) were deemed significantly different from each other. The fourth row illustrates the mean walking-coherence (over time and frequency) for regions of significant coherence in each of the six 10-min periods. Walking-coherence and walking-gain decreased over the duration of the 60-min period. Error bars are the 95% confidence intervals of the mean. The decrease in mean walking-gain was fit with a decaying exponential (black line) and the arrow indicates the exponential’s time constant, which was 67 min. Mean coherence and gain were calculated only for times and frequencies in which coherence was significantly different from zero in the first 10-min time-period using a 95% confidence limit based on the number of strides contributing to the coherence estimate. *m* minutes.

To broadly determine if stimulus related vestibular response attenuation occurs during walking, we compared coherence and gain magnitude in the first 10 min (early) of the 60-min walking period to the last 10 min (late) of the 60-min walking period from the *Walking Treatment* group. Like the pre and post-standing trials, regions of significant stimulus-medio-lateral force walking coherence decreased (mean coherence-early: 0.04 ± 0.02, late: 0.02 ± 0.01) over the hour of walking (Fig. 5). However, unlike standing, the decrease in walking coherence was also accompanied by a significant decrease in walking gain (mean gain-early: 2.72 ± 0.77 N/mA, late: 1.67 ± 0.51 N/mA, overall 38 ± 0.14% decrease) without a clear increase in RMS forces (Fig. 4B). To establish the time course of this response attenuation we fit the decay in gain during walking over time with an intercept only model (AIC: 5.50, BIC: 5.99), a linear model (AIC: − 12.14, BIC: − 11.17), and an exponential model (AIC: − 14.17, BIC: − 13.20) and the exponential model was the best fit (parameters for the best fit are shown in Table 1 lower half). The decay time constant of the exponential model was approximately 67 min. These results suggest that stimulus-related vestibular response attenuation indeed occurs during walking with stimulation, but to a greater degree than during standing, and with a much longer time constant. Surprisingly, the response attenuation observed during walking was not evident in the surrounding pre and post-standing trials, suggesting that mechanisms associated with walking contribute to this process. However, it is important to note that if carry over, or an additional bout of rapid response attenuation, was small, this study may have been underpowered to detect it in the post-standing trials.

## Discussion

We sought to determine whether multiple mechanisms of stimulus-related vestibular response attenuation act to decrease behavioral responses to GVS. We found that the extent and rate of vestibular response attenuation is task dependent. During standing there was an 18% decrease in mean gain, with an approximate 19-s time constant, that exhibited asymptotic behavior after the first 40 s of exposure to the stimulus. A second exposure to the stimulus during standing an hour later did not appear to result in further decreases in gain. In contrast, vestibular response attenuation continued while subjects walked for one hour, resulting in a 38% reduction in mean gain, with a 67-min time constant. This walking-related response attenuation did not appear to carry over to the standing period immediately following walking suggesting that it may occur via, at least, two distinct mechanisms, one of which appears walking-related.

Previous reports of stimulus-related vestibular response attenuation to GVS describe different behaviors over time^12–17^. In some cases, stimulus-related response attenuation asymptotically stabilizes quickly and can be limited in successive trials if a 40-s preliminary exposure to the sinusoidal stimulus is provided^12,13^. We found there to be an 18% decrease in vestibular gain over the first 40 s of exposure to the stimulus during standing, corroborating these previous observations. Others have reported that stimulus-related response attenuation continues over repeated exposures to a pseudorandom stimulus^15,17^. Much like these previous studies, we observed a clear reduction in vestibulomotor gain over the duration of exposure to the stimulus while participants walked, resulting in a roughly 9% decrease in mean gain every 10-min, or a 38% reduction in mean gain over the entire 60-min period. Stimulus-related vestibular response attenuation during walking exhibited a 67 min time constant (compared to 19 s during standing) and it did not appear to carry over into the standing period following walking. The different time constants and the absence of carry over between tasks (i.e. walking-to-standing) suggest that two separate mechanisms may underlie these distinct periods of response attenuation.

Habituation, and more generally stimulus-related response attenuation, is a widely reported phenomena, and yet the mechanisms that underlie such attenuation to GVS in humans remain unclear. GVS is known to modulate vestibular afferent firing rate^34–36^ by bypassing the sensory transduction mechanics, but this does not preclude a peripheral source for such attenuation. One possibility is that vestibular afferent sensitivity to the electric current is modified over time either through mechanisms intrinsic to the afferents or through a feedback process like the efferent vestibular system. Alternatively, central mediation has also been proposed to subserve vestibulo-ocular reflex habituation^37–39^ and may similarly contribute to the observed stimulus-related response attenuation during both standing and walking, albeit by potentially separate mechanisms. The apparent association of one form of stimulus related response attenuation with the rhythmic motor activity of walking may suggest a role for motor prediction that could involve some form of sensory reweighting^40^. Such mechanisms have shown broad utility in motor control^41–43^ and a convergence of such sensory and motor signals has been observed in the cerebellum^44^ where there is evidence of habituation in nearby vestibular nucleus neurons^45^. While it is unclear whether the stimulus-related response attenuation that occurs during walking is cerebellar in origin, given the cerebellum’s known adaptive role in sensory and motor processing^44,46–48^, coupled with its proposed role in habituating the vestibulo-ocular reflex, it seems a leading candidate to play a primary role in attenuating vestibular influence on postural control.

While the anatomical locus for GVS related response attenuation is unclear, one possible explanation for such attenuation during walking is that it shares a mechanism similar to that observed following prolonged periods of passive stochastic motion. Recent research has found that following 10 min of passive stochastic whole-body motion the size of GVS-induced force responses to square-wave GVS stimuli during standing are reduced^49^. Importantly, in this study the largest peak of the GVS-induced force response following passive motion was significantly smaller than it was prior to passive stochastic motion. This finding directly conflicts with our observation that stimulus-related response attenuation during walking does not carry over to the post-walking-standing period. One potential reason for the differences between these two studies is that while walking on a stable surface, much of the observed head variance is predictable and is likely treated differently than unpredictable passive stochastic motion. The latter motion may require generalized attenuation whereas the former can be selectively attenuated^50,51^. Since some component of the head variance during walking is unpredictable, like the stochastic motion, and the random-waveform GVS acts, at least to some degree, like an unpredictable motion signal, it is possible that the mechanisms underlying the attenuated vestibular influence following passive stochastic motion also operate during walking with random-waveform GVS. At this point, however, more evidence is necessary to conclude that the mechanisms underlying vestibular attenuation during passive stochastic motion explain the majority of stimulus-related attenuation observed during walking with GVS.

Over the duration of this study, participants assigned to the *Walking Treatment* and *Walking Control* condition completed 8-min of standing and 60-min of walking. In the *Walking Treatment* condition, participants also had to overcome the small disturbances originating from the stimulus. Given the nature and duration of the study, it is possible that participants experienced a level of fatigue that may have influenced the increase in non-stimulus coupled force variability observed in post-standing trials. Previous research has shown that fatigue can, in some cases, increase center of pressure and sway variability^52,53^. Since we did not take measures of participant fatigue, it is difficult to draw conclusions as to whether fatigue contributed to this increased variability. On the other hand, several researchers have suggested that spontaneous sway may arise as a consequence of vestibular noise^54–57^ and it is possible that vestibular noise could have increased over the duration of this study. Based on the principles of multisensory integration^58^, such an event could result in decreased vestibular efficacy over time, observable through an increase in reliance on non-vestibular cues. If this were the case, one could presumably predict a carry-over effect similar to that observed following stochastic passive motion^49^. However, such carry over was seemingly absent in the results of this study. Considering these alternative explanations, future work is necessary to clarify the mechanisms underlying the observed increase in variance following walking for an hour.

The observation of significant walking-related response attenuation warrants special consideration for studies or interventions using repeated or prolonged exposure to random-waveform GVS during walking. Care should be taken to account for the effect of stimulus-related response attenuation when comparing locomotor trials collected within a single session or across multiple sessions. Similar caution may also be warranted for studies or interventions involving lower amplitude vestibular stimuli, such as the ‘noisy’ stimuli used to reduce sway that have gained recent popularity for review^59,60^. Further research is necessary to determine whether stimulus-related response attenuation could decrease the effectiveness of such stimuli when used for extended periods of time. Lastly, given the small rapid response attenuation observed during standing, a 40-s pre-data collection conditioning stimulus, as proposed by Balter et al.^61^, should be considered in some circumstances to avoid the confounding effect of first exposure response attenuation on comparisons with the remainder of trials.

### Limitations

First, we have implied here that the stimulus-related response attenuation observed during standing is reflective of what is to be expected during direct exposure to the stimulus without concurrent rhythmic motor action. However, it is unclear whether similar response attenuation, with a 19 s time constant, would be observed in other postural contexts, such as long-term stimulus exposure during sitting, or whether it occurs independent of the postural task. Future research should further investigate GVS related response attenuation’s dependence on task. Second, we observed no noteworthy changes in variability during walking. It is possible that small changes in variability over time are present but have been subsumed by the normal stride-to-stride variability. Lastly, the best-fit model for the decay in gain during walking given our data was an exponential model, however since it appears gain may continue to decrease after 60-min future research is necessary to confirm the exponential nature of this decrease.

## Conclusion

We found evidence for two supra-threshold vestibular stimulus-related attenuative mechanisms. First, an initial rapid stimulus-related response attenuation occurring during standing with a 19-s time-constant, resulting in an 18% decrease in vestibular gain. Second, a walking associated response attenuation mechanism with a 67-min time-constant, resulting in a 38% decrease in mean vestibulomotor gain over an hour of stimulation. The response attenuation observed during walking did not appear to carry over to standing. The diverging behavior between response attenuation observed during standing and walking suggests two separate mechanisms act to suppress vestibulomotor gain, one of which appears dependent upon concurrent rhythmic motor activity. Stimulus-related response attenuation could therefore significantly impact research or interventions using prolonged or repeated random-waveform GVS during locomotion.

## Supplementary Information

Supplementary Information.

## Data Availability

The dataset generated and analyzed in this study are available in Utah State University’s Data repository at 10.26078/beqj-ye69.
