# Supplementary material for: Vestibular attenuation to random-waveform galvanic vestibular stimulation during standing and treadmill walking
Source: Sci Rep. 2021 Apr 14;11:8127. doi: 10.1038/s41598-021-87485-4 (PMC8046779; doi:10.1038/s41598-021-87485-4)
Supplement: Supplementary file 1 — Supplementary Information. [file 41598_2021_87485_MOESM1_ESM.docx]

**Vestibular Attenuation to Random-Waveform Galvanic Vestibular Stimulation during Standing and Treadmill Walking**

Hannan, Kelci B.^1^, Todd, Makina K.^1^, Pearson, Nicole J.^1^, Forbes, Patrick A.^2^, & Dakin, Christopher J.^1^*

^1^Department of Kinesiology and Health Sciences, Utah State University, Logan, USA

^2^Department of Neuroscience, Erasmus University Medical Center, Rotterdam, Netherlands

**Author Contributions**

Conceived and designed the experiments: CJD, PAF, MKT, KBH. Performed the experiments: KBH, MKT, NJP. Analysis of data: KBH, CJD. Interpretation of data KBH, CJD, PAF. Revision of manuscript: All authors

**Corresponding Author**

Dr. Christopher J. Dakin

Department of Kinesiology and Health Sciences,

Utah State University,

Logan, UT, USA.

chris.dakin@usu.edu

Supplementary Methods S1

**EVS Pre-Screening Questionnaire**

This questionnaire is meant to identify participants considered at risk for electric stimulation near the brain. It is modified from Transcranial Magnetic Stimulation screening form^[[1]](#footnote-1),^^[[2]](#footnote-2)^. Your identity, will be kept confidential at all times and stored in locked facilities in the Kinesiology and Health Science Department; only research team members will have access to this information.

**This screening is conducted for your safety; the information provided will not be used for any purpose other than the screening. Please answer all questions honestly. Please contact the Principal Investigator if any question is unclear.**

Name: ___________________________ Sex:__________ Age:______________________

Weight:__________ Height:____________

**Circle answer**

| 1. Do you have epilepsy or have you ever had a convulsion or a seizure? | Yes / No |
| --- | --- |
| 1. Has any of your immediate family members (i.e., blood relatives) been treated for, or suspected of having, epilepsy? | Yes / No |
| 1. Have you ever had an anxiety-induced fainting spell? | Yes / No |
| 1. Have you ever had a head trauma that was diagnosed as a concussion or was associated with loss of consciousness? | Yes / No |
| 1. Have you experienced a period of severe headaches (e.g., migraine) in the past 12 months? | Yes / No |
| 1. Do you have any noise-induced hearing loss or ringing in your ears? | Yes / No |
| 1. Do you have cochlear implants? | Yes / No |
| 1. Are you pregnant or is there any chance that you might be? | Yes / No |
| 1. Do you have metal in the brain, skull or neck (*for example splinters, fragments, clips, etc.*)? | Yes / No |
| 1. Do you have an implanted neurostimulator (*for example a deep brain stimulator, spinal cord stimulator, or vagus nerve stimulator*)? | Yes / No |
| 1. Do you have a cardiac pacemaker or intracardiac lines (wires to monitor your heart)? | Yes / No |
| 1. Do you have an implanted medication infusion device (*for example an insulin pump*)? | Yes / No |
| 1. Have you ever been told you have a heart disease (*for example myocardial infarction, arteriosclerosis, heart disease, heart block, coronary thrombosis, rheumatic heart, heart attack, aneurism, coronary occlusion, angina, heart failure, heart murmur*)? | Yes / No |
| 1. Are you taking any medications? | Yes / No |
| If yes, please list: | |
| 1. Have you recently reduced your average alcohol consumption from severe to (near-)abstinence? | Yes / No |
| 1. Have you ever undergone Vestibular Stimulation in the past?   If so, were there any problems? | Yes / No  Yes / No |
| 1. Have you undergone an MRI of any body part in the past?   If so, were there any problems? | Yes / No  Yes / No |

I hereby declare that all information given on this application is true and complete.

___________________________________________________________________________Participant’s Signature Participant’s Name, Printed Date

1. Rossi, S., Hallett, M., Rossini, P. M., Pascual-Leone, A., & Safety of TMS Consensus Group (2009). Safety, ethical considerations, and application guidelines for the use of transcranial magnetic stimulation in clinical practice and research. *Clinical Neurophysiology, 120*, 2008-2039. [↑](#footnote-ref-1)
2. Rossi, S., Hallett, M., Rossini, P. M., & Pascual-Leone, A. (2011). Screening questionnaire before TMS: an update. *Clinical Neurophysiology, 122*, 1686.  [↑](#footnote-ref-2)
